# Chagas Disease Cardiomyopathy: Immunopathology and Genetics

**DOI:** 10.1155/2014/683230

**Published:** 2014-08-19

**Authors:** Edecio Cunha-Neto, Christophe Chevillard

**Affiliations:** ^1^Heart Institute (InCor), University of São Paulo School of Medicine, Avenida Dr. Enéas de Carvalho Aguiar, 44 Bloco 2 9° Andar, 05406-000 São Paulo, SP, Brazil; ^2^Institute for Investigation in Immunology (iii), INCT, São Paulo, SP, Brazil; ^3^Division of Clinical Immunology and Allergy, University of São Paulo School of Medicine, 05406-000 São Paulo, SP, Brazil; ^4^Aix-Marseille Université, INSERM, GIMP UMR_S906, 13385 Marseille, France

## Abstract

Chagas disease, caused by the protozoan *Trypanosoma cruzi*, is endemic in Latin America and affects ca. 10 million people worldwide. About 30% of Chagas disease patients develop chronic Chagas disease cardiomyopathy (CCC), a particularly lethal inflammatory cardiomyopathy that occurs decades after the initial infection, while most patients remain asymptomatic. Mortality rate is higher than that of noninflammatory cardiomyopathy. CCC heart lesions present a Th1 T-cell-rich myocarditis, with cardiomyocyte hypertrophy and prominent fibrosis. Data suggest that the myocarditis plays a major pathogenetic role in disease progression. Major unmet goals include the thorough understanding of disease pathogenesis and therapeutic targets and identification of prognostic genetic factors. Chagas disease thus remains a neglected disease, with no vaccines or antiparasitic drugs proven efficient in chronically infected adults, when most patients are diagnosed. Both familial aggregation of CCC cases and the fact that only 30% of infected patients develop CCC suggest there might be a genetic component to disease susceptibility. Moreover, previous case-control studies have identified some genes associated to human susceptibility to CCC. In this paper, we will review the immunopathogenesis and genetics of Chagas disease, highlighting studies that shed light on the differential progression of Chagas disease patients to CCC.

## 1. Introduction

Chagas disease (American trypanosomiasis) is caused by the protozoan parasite* Trypanosoma cruzi* and transmitted by the reduviid bug (called “barbeiro” in Brazil) in the poor, rural endemic areas of Latin America. The disease was discovered in 1909 by the Brazilian physician Carlos Chagas. Unfortunately, Chagas disease remains a neglected disease and a contemporary public health concern, with no vaccines available so far and only few antiparasitic drugs only proven efficient for treating the acute phase of the disease—but none has yet been shown to be effective in chronically infected adults, the stage when the great majority of patients are diagnosed. Approximately 8 million people are infected with* T. cruzi* in Central and South America [1]. At least 120 million are at risk from Chagas disease [2]. Chagas disease is a major cause of heart disease and cardiovascular-related deaths in endemic areas located in Latin America and causes significant economical burden in affected countries. Approximately 12,000 deaths attributable to Chagas disease occur annually, typically due to severe chronic Chagas disease cardiomyopathy (CCC) [1], which develops in ca. 30% of infected individuals decades after infection. The only available treatment for end-stage CCC patients is heart transplantation, a high-cost, high complexity intervention which is not available in a timely fashion for the majority of patients [3]. Chagas disease is now a global health issue. Thirteen million persons have migrated from endemic countries to the United States, and it is estimated that 0.3–1 million of which have chronic* T. cruzi* infection [4]; the European Community has also received large numbers of migrant from endemic areas. The World Health Organization estimates that 56 thousand new cases of Chagas disease occur every year [1, 3].

## 2. Natural History and Pathogenesis

The natural history of Chagas disease includes an acute and a chronic phase. The high parasite load typical of acute* T. cruzi* infection is dampened by the immune response into a low-grade chronic persistent infection [5]. CCC is an inflammatory cardiomyopathy that affects approximately 30% of infected individuals and occurs 5–30 years after acute infection, while the remaining patients develop digestive disorders (5–10%) or remain asymptomatic and free from cardiac or digestive disorders, (60–70%) the indeterminate phase (ASY). Approximately 1/3 of the CCC patients (or 10% of infected patients) develop a particularly lethal form of dilated cardiomyopathy (severe end-stage CCC) with ventricular dysfunction, heart failure, and arrhythmia. Clinical severity is correlated with the occurrence of myocarditis. ASY patients display minimal myocardial inflammation while patients with severe, end-stage CCC display frequent and intense myocarditis; moderate CCC patients display an intermediate level of myocarditis [6]. Our group has found a positive correlation between the cellularity of the infiltrate and degree of ventricular dilation in the Syrian hamster model of dilated Chagas disease cardiomyopathy with chronic* T. cruzi* infection ([7, 8] and data not shown). Survival in severe CCC is significantly shorter than clinically similar cardiomyopathies of noninflamammatory etiology, like idiopathic dilated cardiomyopathy (DCM) [9, 10]. Taken together, current literature suggests that myocarditis plays a major role in cardiomyocyte destruction, fibrosis, and disease progression [6, 11].

Histologically, CCC myocardium displays a diffuse myocarditis with foci of inflammatory infiltrate and heart fiber damage, prominent fibrosis, and scarcity of* T. cruzi *parasites (reviewed in [12]). The inflammatory infiltrate of CCC heart lesions is composed mainly of T cells displaying a Th1-type cytokine profile (2 : 1 ratio of CD8+/CD4+ T cell ratio) and macrophages [11, 13–17]. The list of cytokines and chemokines found to be increased in CCC is in Table 1. Chronic myocardial inflammation in CCC may be secondary to recognition of either* T. cruzi* antigen/DNA deposited/detected in hearts of both CCC and ASY patients [18] or myocardial antigens. Our group has identified both* T. cruzi*-specific [19] and* T. cruzi*-cross-reactive cardiac-myosin-specific T cells [20] in the myocardial inflammatory infiltrate, thus reactive to antigens that can be found in hearts of all Chagas disease patients.

## 3. Immunological Dynamics during the Acute and Chronic Phases of* T. cruzi* Infection

Shortly after the acute infection starts,* T. cruzi* components, including its DNA and membrane glycoconjugates, trigger innate immunity via Toll-like receptors in macrophages and dendritic cells, among other cell types [21]. Upon activation, such cells secrete proinflammatory cytokines and chemokines, express costimulatory receptors, and increase endocytosis and intracellular killing of parasites through release of reactive oxygen and nitrogen species. Released cytokines further activate other inflammatory cells [22, 23]. Macrophages and dendritic cells that have endocytosed the parasite subsequently elicit a strong T cell and antibody response against* T. cruzi*. IFN-*γ*-producing* T. cruzi*-specific T cells are thus generated [23], which migrate to sites of* T. cruzi*-induced inflammation, including the myocardium, in response to chemokines [24, 25] and blood parasitism. Silva et al. observed in a murine model of Chagas disease that CD8+ IFN*γ*+Perforin−T cells correlated with a less intense cardiac damage, whereas CD8+ IFN*γ*-Perforin+ T cells correlated with tissue damage 120 days postinfection. On the other hand, IL-10 and TGF-*β* are associated with susceptibility to* T. cruzi* infection in mice [26–28]. Recent data show that IL-17 and CD4+CD25+GITR+Foxp3+ regulatory T cells control the parasite-induced myocarditis and resistance to* T. cruzi *infection in mice [29, 30]. Patients with the acute phase of Chagas disease display increased circulating levels of IL-6 and TNF-*α* [31] and increased production of IFN-*γ* by mononuclear cells [32].

Th1/proinflammatory cytokines are also produced along the chronic phase* T. cruzi* infection, both in infected mice and in Chagas disease patients. Increased levels of plasma TNF-*α* and peripheral blood mononuclear cell-produced IFN-*γ* are detected in CCC and even in ASY patients [15, 33, 34], probably as a response to parasite persistence. Patients who develop Chagas cardiomyopathy display a particularly strong Th1-type immune response as compared to ASY patients. CCC patients show an increased number of CD4+ and CD8+ IFN-*γ*-producing T cells in the peripheral blood, with reduced numbers of IL-10-producing CD4+CD25+ regulatory T cells [15, 35, 36] and CD4+CD25+ FoxP3+ regulatory T cells [37] as compared with patients in the ASY form of Chagas disease. Taken together, this suggests that regulatory T cells may play a role in the control of the intensity of inflammation in chronic Chagas disease.

The exacerbated Th1 response observed in the peripheral blood of CCC patients is reflected on the Th1-rich inflammatory infiltrate predominantly secreting IFN-*γ* and TNF*α*, with lower production of IL-4, IL-6, IL-7, and IL-15 found in their heart tissue as evidenced by immunohistochemistry and mRNA expression studies [13–15, 17, 38–40]. Further to corroborate this, we recently observed significant expression of the hallmark Th1 transcription factor, T-bet, in the CCC myocardium (unpublished observations). Conversely, mRNA expressions GATA3, FoxP3, and ROR*γ*T, hallmark transcription factors of Th2, Treg, and Th17 populations, and their signature cytokines and molecular markers were low or undetectable [41]. This is in line with the reduced number of FoxP3+ cells in CCC myocardial tissue [37]. These results suggest the predominant Th1 infiltrate in CCC myocardium is essentially unopposed and suffers little regulation, which could explain its destructiveness, most likely due to excessive collateral damage by type-1 CD8 T cells as described for* T. cruzi*-infected mice [42]. We hypothesized that the selective accumulation of Th1 T cells in CCC myocardium at the expense of other T cell types could be a result of an imbalance at the Th1-associated chemokine-chemokine receptor axes. We were able to detect mononuclear cells that express CXCR3, CCR5, CCR4, CXCL9, and CCL5 in the myocardium of CCC patients using confocal immunofluorescence, and real time qPCR analysis also showed increased mRNA expression of CCR2, CXCR3, CCR5, CCR4, CCR7, and their main chemokine ligands, including the monocyte-chemoattractant chemokine CCL2. CCL5 and CCL9 were the most upregulated chemokine genes in CCC heart tissue. Significantly, the intensity of the myocardial infiltrate was positively correlated with CXCL9 mRNA expression [38, 41]. These results were consistent with a major role of locally produced Th1-chemoattractant chemokines in the accumulation of Th1 T cells in CCC heart tissue.

In recent years, new-generation high-throughput or “omics” technologies have been widely applied in solving complex biological problems, by measuring multiple components simultaneously, in a data-driven, hypothesis-generating, large-scale research model. Systems biology approaches yield data that identify metabolic or signaling pathways involved in the pathogenesis of particular diseases, identifying therapeutic targets, as well as diagnosis and prognosis markers. Our group has used the “omics” approach to elucidate the pathogenesis of human CCC, especially the downstream events occurring in myocardial tissue that could be a consequence of Th1 T cell-driven myocardial inflammation. Cunha-Neto et al. analyzed the gene expression profiling of myocardial tissues of CCC, the noninflammatory idiopathic dilated cardiomyopathy (DCM), and heart donors with a cDNA microarray based on genes expressed in cardiovascular tissue [38]. Immune response-related genes were upregulated only in CCC patients. Multiple IFN-*γ*-inducible genes were strongly upregulated, indicating prominent IFN-*γ* signaling that included cardiomyocyte genes. We subsequently observed that IFN-*γ* and CCL2 treatment of cultured cardiomyocytes induced a strong increase in expression of atrial natriuretic factor (ANF) mRNA, a key member of the embryonic/hypertrophic cardiomyocyte gene expression program. Accordingly, although myocardium ANF mRNA levels were elevated in both disease groups, CCC myocardium expressed 6-fold higher ANF mRNA levels than DCM [37, 41]. The connection between myocardial production of IFN-*γ* and cardiomyocyte gene expression changes leading to hypertrophy, ventricular dilation, and heart failure was previously unknown and unexpected and was only identified because of the use of a data-driven “omics”/systems biology approach. It is known that other inflammatory mediators such as TNF-*α*, CCL2 [38], IL-18, CCL21, and phosphorylated Smad2 involved in TGF*β* signaling [43], upregulated in CCC myocardium, are able to induce cardiomyocyte hypertrophy and fibrosis [44, 45]. Taken together, data suggest that locally produced inflammatory mediators have nonimmunological effects on myocardial tissue distinct from direct tissue damage, which may play a significant pathogenic role in CCC, by modulating gene and protein expression in pathways essential to the development of CCC. Gene expression profiling also disclosed that lipid metabolism and mitochondrial oxidative phosphorylation genes were selectively modulated in CCC myocardial tissue, suggesting a specific energy imbalance. Using proteomic analysis (bidimensional electrophoresis and MALDI-TOF mass spectrometry), our group has established a proteomic inventory of >100 distinct myocardial proteins in CCC myocardial tissue [46]. Preliminary data from differential protein expression analysis in the myocardium of CCC, DCM, and ischemic cardiomyopathy (IC) patients as compared to control heart donor myocardial samples allowed the identification of prominently altered pathways, including cardiac remodeling and depressed energy metabolism. Validation studies have shown that both total creatine kinase activity and ATP synthase alpha chain protein levels were significantly lower in CCC samples than IC, IDC, and control samples [47], corroborating the results of the transcriptomic and proteomic analysis. It has been shown that IFN-*γ* may reduce expression of creatine kinase, which could be a possible mechanism for our findings in CCC hearts (reviewed in [12]). On the other hand, the finding that different degrees of myocardial inflammation were found in clinically similar end-stage CCC patients [41] suggests that noninflammatory factors, probably related to the heart itself, could potentially play a determinant role in disease progression. This is reinforced by findings on the Syrian hamster model of dilated Chagas cardiomyopathy. While the intensity of inflammation is correlated with ventricular dilation (i.e., disease progression), it is not different among animals who die from congestive heart failure or survivors ([7] and data not shown). This may suggest that additional, noninflammatory factors could contribute to severity or progression to death from CCC. Indeed, myocardial resilience, or the ability of myocardial tissue to withstand inflammatory and other stress, may be a key factor. One possible example of a myocardial resilience factor involves susceptibility to apoptosis. Some congenital heart diseases are associated with low levels of cardiomyocyte alpha-cardiac actin, which is related to increased susceptibility to apoptosis [48]. Apoptosis-inducing cytokines, abundant in end-stage CCC myocardium, will likely induce increased apoptosis in cardiomyocytes expressing low levels of cardiac actin. Significantly, we found that myocardial levels of cardiac actin protein are reduced in CCC myocardium. Indeed, Tostes et al. demonstrated the occurrence of significantly higher than normal levels of cardiomyocyte apoptosis in myocardial tissue from severe CCC cases [49]. It is also conceivable that reduced levels of alpha-cardiac actin could impact on Z-disc mechanosensor function and thereby enhance cardiomyocyte apoptosis. In a disease setting, this could accelerate progression to heart failure [50]. We thus consider that progression of chronic Chagas disease may be a result of multiple factors occurring in the affected myocardium including (i) the intensity of the myocardial inflammation, (ii) direct inflammatory damage, (iii) inflammatory mediator-induced changes in myocardial gene and protein expression, and (iv) the ability of the myocardial tissue to withstand inflammatory and other stress.

## 4. Genetic Polymorphisms and Susceptibility to CCC Development

Mechanisms underlying differential progression to CCC are still incompletely understood. Familial aggregation of CCC has been described [51], suggesting that there might be a genetic component to disease susceptibility [51]. This is also supported by the fact that only one-third of* T. cruzi*-infected individuals develop CCC. A possible role of polymorphisms is that the genetically heterogeneous* T. cruzi* parasite itself in disease outcome cannot be ruled out. CCC patients display a more intense inflammatory response than the asymptomatic patients, who seem to have a more regulated immune response. Given the importance of inflammatory mechanisms for CCC pathogenesis, genetic susceptibility to CCC may result from functionally relevant genetic polymorphisms that lead to variations in the intensity of the innate or acquired immune response and in inflammatory cytokines and chemokines involved in the pathogenesis of the disease.

A number of case-control genetic studies have found signs of association between gene polymorphisms and disease progression. Polymorphisms in the HLA class I and class II loci have been especially investigated in association with chronic Chagas cardiomyopathy. In a Venezuelan cohort Fernandez-Mestre et al. demonstrate the first evidence of association between Chagas disease and HLA genetic susceptibility when they analyzed HLA class II alleles in a sample of 67 serologically positive individuals with and without cardiomyopathy and compared with 156 healthy controls of similar ethnic origin [52]. The comparison of DRB1 and DQB1 allele frequencies among the patients and healthy control subjects showed a decreased frequency of DRB1∗14 and DQB1∗0303 in the patients, suggesting independent protective effects to the chronic infection in that population. Allele frequencies comparison between patients with and without cardiomyopathy showed a higher frequency of DRB1∗01, DRB1∗08, and DQB1∗0501 and a decreased frequency of DRB1∗1501 in the patients with arrhythmia and congestive heart failure [52]. These results suggest that HLA class II genes may be associated with the development of a chronic infection and with heart damage in Chagas disease. At the same time, Deghaide et al. have characterised a Brazilian population including 176 patients presenting with pure cardiomyopathy with heart failure (*N* = 60), cardiomyopathy without heart failure (*N* = 18), pure digestive tract manifestations (*N* = 25), cardiac plus digestive disease (*N* = 40), and asymptomatic patients with positive serology for chronic* T. cruzi* infection (*N* = 33) and noninfected individuals (*N* = 448). Serologic HLA class II analysis showed that HLA-DQ1 conferred susceptibility to while HLA-DQ7 antigen conferred protection against the development of the disease in the total group of patients. Oligonucleotide typing has shown that HLADQB1∗06 alleles were underrepresented in the total group and in the subgroups presenting with pure digestive or cardiac disease, conferring closely similar relative risks and preventive fractions. Asymptomatic patients showed a significant increase of HLA-DQB1∗0302 specificity [53]. Layrisse et al. have shown a strong association of HLA class I gene (HLAC∗03 allele) with CCC as compared with asymptomatic subjects in a Venezuelan cohort (the same population as Fernandez-Mestre et al.) [54]. After increasing their cohort (35 asymptomatic cases and 72 symptomatic cases), the authors confirmed their previous results and they have shown that the DPB1∗0401 allele frequency is also significantly increased in patients with heart disease while DPB1∗0101 frequency is higher among the asymptomatic group compared with CCC patients [55]. The results on DRB1 and DQB1 were partially confirmed on a Peruvian and Argentinean population [56, 57], whereas this replication failed on a Brazilian population [58]. Taken together, results suggest that HLA class II association may vary with the genetic background of the studied population, perhaps indicating differential linkage disequilibrium with functional variants in different populations. Genes in the MHC class III/*TNFA* region were also probed for association with Chagas disease progression. Association was detected with polymorphism* TNFA-308A/G* in a Mexican population (27 asymptomatic subjects, 27 patients with chronic cardiomyopathy, and 169 healthy individuals) [59]. Similarly, Campelo et al. have conducted an evaluation of genetic susceptibility to chronic disease in relationship of five microsatellite polymorphisms in and around a series of Brazilian chagasic patients stratified according to the clinical form of disease presentation, that is, cardiac, digestive, digestive plus cardiac, or indeterminate form (54 patients with cardiomyopathy with heart failure, 17 patients with cardiomyopathy without heart failure, 25 patients with pure digestive manifestations, 33 patients with digestive plus cardiac manifestations, 33 other patients characterized by indeterminate form, and 221 negative serology subjects). The relative risks associated with the susceptibility alleles ranged from 1.674 to 10.21, indicating that the individuals who possess these susceptibility alleles have almost 2 to 10 times higher risk of developing a given form of chronic disease if infected [60]. Twelve haplotype frequencies revealed significant differences when patients were considered as a whole or stratified according to the clinical variant and were compared to controls. All these results suggest that this chromosomal region is associated with susceptibility to or resistance against CCC forms. Even though this result was not confirmed into two independent studies performed on Peruvian [61] and Brazilian populations [62] a meta-analysis with data from the three studies disclosed a significant association on the* TNFA*-308 polymorphism with disease progression (Table 2). This indicates that this chromosomal region may indeed be involved in the genetic control of susceptibility to CCC development. The discrepancies between studies could have been due either to modest clinical group size or to differing genetic backgrounds, where the functional variant could be in strong linkage disequilibrium with the* TNFA*-308 polymorphism only in the Mexican population. Drigo et al. [63] have shown that end-stage CCC patients carrying* TNFA*-308A and/or TNFa microsatellite polymorphisms display a significantly shorter survival time compared to those carrying other alleles (166 CCC patients compared to 80 asymptomatic individuals employed as control group). Ramasawmy et al. have also focused their efforts on three other genes (UAP56, LTA, and IKBL) located into the HLA class III region, in proximity to the* TNFA* locus. For the polymorphism UAP56-22G/C, a significant difference in frequency between 154 Brazilian CCC patients and the 76 Brazilian asymptomatic patients was revealed at the genotype level [64]. The UAP56-22C allele seems to confer susceptibility to CCC. Lymphotoxin-*α* protein (encoded by LTA gene) is a proinflammatory cytokine which also induces adhesion molecules and cytokines from vascular endothelial cells and vascular smooth-muscle cells, which may contribute to the inflammation process. The LTA+80C/C genotype was significantly more common in CCC patients than asymptomatic patients [65]. This result was confirmed later on an independent Brazilian cohort [66]. A previous study done on hepatitis C virus infection has showed that a non-HLA gene block within the MHC class III region, including the IKBL, ATP6V1G2, BAT1, MICB, and MICA genes, was strongly associated with development of dilated cardiomyopathy. Among these genes, IKBL encodes an inhibitor of NF-*κ*B which plays a key role in innate immunity. Ramasawmy et al. provide evidence that two variants (IKBL-62A/T and IKBL-262A/G) in the promoter region of the IKBL gene are associated with susceptibility to develop CCC [67]. Similar trend was observed for the IKBL-262A homozygotes. A haplotype analysis led to the identification of a susceptibility haplotype (IKBL-262A IKBL-62A) more frequent in CCC patients [67].

On another side, polymorphisms in additional cytokine genes have been also investigated. On a Brazilian population, including hundred fifty-five patients in the chronic phase and 43 individuals without Chagas disease, the polymorphism IL10-1082G/A, which correlates with lower expression of IL-10, was associated with the development of Chagas disease cardiomyopathy [68]. An independent study including 104 CCC patients and 60 noninfected controls suggests that an epistasis between MHC and IL-10 is associated with susceptibility/resistance to Chagas disease [69]. The interleukin 1 (IL-1) is a primary inflammatory cytokine and has been implicated in mediating both acute and chronic pathologic inflammatory diseases [70]. A Colombian study (130 patients with cardiomyopathy and 130 asymptomatic subjects) has detected significant differences in the distribution of the* IL1B* +5810 genotypes between cardiomyopathic patients and asymptomatic individuals [71]. A haplotype covering the IL1A, IL1B, and IL1RN genes was associated with protection [71]. Putative association for the IL1 antagonist gene (IL1RN) was described on a Mexican population (58 CCC patients, 28 asymptomatic, 50 seronegative individuals with idiopathic dilated cardiomyopathy, and 109 healthy individuals) [72]. Interleukin-12 (IL-12) induces production of interferon-*γ* (IFN-*γ*) and favours the differentiation of T helper 1 (Th1). Different studies corroborate the crucial role of IL-12 in host resistance to* T. cruzi* infection [73–75]. The authors observed that the IL12+1188C allele was present at significantly higher frequency in the CCC population than in asymptomatic (Colombian population including 200 seronegative individuals, 260 serologically positive patients, 130 with Chagas cardiomyopathy, and 130 asymptomatic) [76]. However, this polymorphism does not discriminate the seropositive patients from the control individuals [76].

The TLRs play an important role in innate immunity by acting as sensors for invading pathogens. The intracellular signaling of TLRs is mediated by at least 5 adaptor proteins, including MyD88, MAL/TIRAP, TRIF, TICAM, and SARM [77]. On a Brazilian population including 169 patients with cardiomyopathy and 76 asymptomatic individuals, the frequency of homozygosity for the* MAL/TIRAP *180S allele was significantly higher among patients with CCC than among asymptomatic patients, whereas the percentage of subjects homozygous for the* MAL/TIRAP *180L allele was similar in both groups. The percentage of subjects heterozygous for* MAL/TIRAP *S180L among patients with CCC was significantly different from the percentage found in asymptomatic patients [78]. Migration inhibitory factor (MIF) protein is a pleiotropic cytokine produced by activated T cells, macrophages. For the polymorphism, MIF-173G/C, a statistically significant difference was detected between patients and controls in the Colombian cohort (240 chagasic patients and 199 controls). A similar association was found in the Peruvian cohort (74 chagasic patients and 85 controls). A meta-analysis has demonstrated that the MIF-173C allele confers a risk effect in Chagas patients. Moreover, a dose effect for the susceptibility allele was observed [79]. Locally produced chemokines such as CCL2, CCL5, and CXCL9 play a major role in inflammatory cell recruitment to the heart in* T. cruzi*-induced acute myocarditis in mice as well as chronic human Chagas disease myocarditis [24, 41, 80, 81]. Cytokine-mediated production of CCL2 by cardiomyocytes seems to participate in the killing of* T. cruzi *through a nitric oxide-dependent mechanism [82, 83]. Ramasawmy et al. showed an association between the* CCL2*-2518A/G polymorphism and CCC development [84]. Calzada et al. observed, on seropositive and seronegative Peruvian individuals, that the CCR5 59029A/G genotype was significantly increased in asymptomatic compared to CCC patients (85 seropositive and 87 seronegative individuals) [85]. An independent study, performed in Venezuela, indicates that a distinct genotype in the CCR5 gene is also associated with Chagas disease (asymptomatic versus arrhythmic and cardiomyopathic) [86]. Our group has found that the CCR5 rs1799988 CC genotype was significantly more frequent in the severe CCC than with the moderate CCC group, although polymorphisms in the CCL4, CCL5, CCL17, CCL19, and CXCR3 genes, all displaying upregulated expression in CCC myocardial tissue, were not associated with CCC [41].

Some functional polymorphisms have been identified. On a Brazilian population, including 149 asymptomatic subjects, 79 moderate CCC patients (EF > 40%), and 95 severe CCC patients (EF > 40%), our group has reported that chemokine gene polymorphisms* CXCL9* rs10336* CC* and* CXCL10* rs3921* GG* were significantly less frequent among the severe CCC patients with ventricular dysfunction as compared to moderate CCC patients in a Brazilian cohort study [41]. We then observed that myocardial explants of end-stage CCC patients submitted to heart transplantation carrying the lower risk genotypes displayed a 2–6-fold reduction in mRNA expression of CXCL9 and CXCL10 as compared to those with other* CXCL9/CXCL10* genotypes. However, only the* CXCL9 rs10336 GG* polymorphism was associated with reduced intensity of myocarditis; significantly, only myocardial mRNA levels of CXCL9 were strongly associated with intensity of myocarditis. Taken together, our results may suggest that genetic polymorphisms affecting local expression of CXCL9 and possibly CXCL10 control the intensity of myocarditis through modulation of CXCL9 and/or CXCL10 expression in the myocardium and the ensuing effects on the local expression of chemokine ligands of the CXCR3/CCR5/CCR4/CCR7. Considering that polymorphisms at the immunoregulatory CTLA-4 and PDCD1 genes may alter their inhibitory function, we investigated the association of alleles, genotypes, and haplotypes of polymorphic sites observed at the CTLA-4 and PDCD1 genes with different clinical manifestations of chronic Chagas disease (indeterminate, cardiac, digestive, and mixed). Dias et al. conduct a study on Brazilian subjects (90 Chagas disease patients with cardiac forms, 67 Chagas disease patients with digestive form, 39 Chagas disease patients with cardiac and digestive asymptomatic subjects, and 326 noninfected controls). They showed that alleles, genotypes, and haplotypes reported to increase the expression of the regulatory molecule CTLA-4 were associated with the indeterminate form of the disease [87].

Several studies on candidate genes putatively involved in the control of* T. cruzi* infection or myocardial function failed to display an association with CCC. This was reported for the TLR1, TLR2, TLR4, TLR5, TLR9 [88], PTPN22 [89], NRAMP1 [90], ACE [91], NOS2 [92], CCL4, CCL5, CCL17, CCL19, and CXCR3 [41], beta-cardiac myosin heavy chain [58], IL4 [93], and IFNG [94]. Such lack of association or fault positive association may be due either to the limited number of tested SNPs or to the low statistical power owing to limited cohort size. In order to avoid this bias, it is essential to set up large-scale genetic studies using new technologies on large study populations with clearly established phenotypes. In this way, we have enrolled a large Brazilian population and we performed a replication study (Brazilian population, 315 CCC patients and 118 asymptomatic individuals). Our genetic analysis focused on CCR5, CCL2, and MAL/TIRAP genes. The CCL2rs2530797A/A and TIRAPrs8177376A/A were associated with an increased susceptibility whereas the CCR5rs3176763C/C genotype is associated with protection of CCC [95]. Our data show that polymorphisms affecting key molecules involved in several immune parameters (innate immunity signal transduction and T cell/monocyte migration) play a role in genetic susceptibility to CCC development. This also points out to the multigenic character of CCC, each polymorphism imparting a small contribution [95]. In addition, recent results from our groups showed that a single nucleotide polymorphism in the promoter region of the alpha-cardiac actin gene (*ACTC1*) associated with CCC influences transcription factor binding, implying that the polymorphism may influence myocardial transcriptional levels of the highly relevant ACTC1 gene [96]. These results were obtained on a Brazilian population including 315 CCC patients and 118 asymptomatic individuals, and the same trend of association was found on a second independent cohort including 102 CCC patients and 36 asymptomatic individuals.

The first genome-wide association study (GWAS) on Chagas disease was published in 2013 [97]. This analysis included 600 Brazilian* T. cruzi* seropositive blood donors of different clinical forms and 488 Brazilian seronegative donors. Several phenotypes were analyzed, in addition to cardiomyopathy considered as the main trait. Authors also evaluated a limited number of specific parameters, including ejection fraction, PR interval, QRS duration (QRS), corrected QT interval (QTc), EIA signal/cutoff levels, and* T. cruzi* PCR status. Of the 600* T. cruzi* seropositive donors cases, 221 were classified as having CCC, 311 had no cardiomyopathy, and 68 were inconclusive. For cardiomyopathy, two trends of association (after multiple comparison corrections) were detected for markers located around SLCO1B1 gene. SLCO1B1 is a membrane transporter that belongs to a solute carrier family and plays a role in drug metabolism. It is expressed in the liver, brain, heart, and kidney and transports organic anions, such as digoxin, bilirubin, methotrexate, and statins. In addition, loss-of-function mutations may be associated with impaired drug action in target tissues [98]. Moreover, a cluster of 12 SNPs within introns of COL14A1 was associated with PCR positivity. COL14A1 is a fibril-associated collagen which interacts with the fibril surface and regulates fibrillogenesis [99, 100]. Probably all these markers at this locus are in linkage disequilibrium. Furthermore, HSPB8 is a small heat shock protein whose heart specific overexpression induces myocardial hypertrophy [101]. HSPB8-transgenic mice bearing the K141N mutation expressed myocardial hypertrophy, ventricular dysfunction, and apical fibrosis—the latter being a hallmark of heart involvement in CCC [102]. Significantly, expression of HSPB8 is selectively increased in myocardial tissue from CCC patients, rather than in idiopathic dilated cardiomyopathy patients [38]. However, these indications remain suggestive due to the limited size of the studied cohort for a GWAS study. Surprisingly, no polymorphisms in immune-related genes were found.

The use of a systematic approach to identify genes proven to be pathogenetically relevant in CCC could greatly accelerate the finding of functional genetic polymorphisms. This would have a strong impact in the understanding of the pathogenic process in CCC, as well as in diagnosis, prognosis, and therapeutics.

## Figures and Tables

**Table 1:** Cytokine and chemokine expression in Chagas disease and animal models.

Cytokines/chemokines	Phase (acute/chronic/IND/severe/moderate CCC)	Host (mouse/human)	Organ/cell type	Reference
IFN-*γ*	Severe CCC	Human	Mononuclear cells	[15, 35]
IFN-*γ*	Severe CCC	Human	Myocardium	[39, 103]
IFN-*γ*	Severe CCC	Human	Heart-infiltrating T cells	[15]
IFN-*γ*	IND, Severe CCC	Human	Plasma	[15, 33, 81]
TNF-*α*	Severe CCC	Human	Mononuclear cells	[15, 35]
TNF-*α*	Severe CCC	Human	Heart-infiltrating T cells	[15]
TNF-*α*	Severe CCC	Human	Myocardium	[39, 103]
TNF-*α*	IND and Severe CCC	Human	Plasma	[15, 33, 81]
IFN-*γ*	Acute/chronic	Mouse	Heart	[104–106]
TNF-*α*	Acute/chronic	Mouse	Heart	[42]
IL-6	Severe CCC	Human	Heart-infiltrating T cells	[15, 39, 103]
IL-2	Severe CCC	Human	Heart-infiltrating T cells	[15, 39, 103]
IL-4	Severe CCC	Human	Heart-infiltrating T cells	[15, 39, 103]
IL-10	Severe CCC	Human	Heart-infiltrating T cells	[15, 39, 103]
IL-7	Severe CCC	Human	Myocardium	[40]
IL-15	Severe CCC	Human	Myocardium	[40]
IL-12	Acute	Mouse	Mononuclear cells	[75]
IL-18	Acute	Mouse	Mononuclear cells	[107]
IL-10	Acute	Mouse	Mononuclear cells	[26–28]
TGF-*β*	Acute	Mouse	Mononuclear cells	[26–28]
IL-17	Chronic	Mouse	Mononuclear cells	[29]
CCL2, CXCL10, CXCL9 (mRNA)	Severe CCC	Human	Myocardium	[38]
CCR2, CXCR3 (mRNA)	Severe CCC	Human	Myocardium	[38]
CCR5, CXCR3	Severe CCC, IND	Human	Mononuclear cells	[108]
CCL5, CXCL9, CXCL10	Chronic	Mouse	Cardiomyocytes	[109]
CCR5	Chronic	Mouse	Heart	[25, 83]
CCL5, CCL4, CXCR3 (mRNA)	Chronic	Dog	Heart	[110]

**Table 2:** Meta-analysis on the *TNFA*-308 polymorphism.

Study name	Statistics for each study			
	*P* value	Odds ratio	Lower limit	Upper limit
Rodríguez-Pérez et al. [59]	0.006	3.03	1.376	6.671
Drigo et al. [62]	0.701	1.129	0.608	2.097
Beraún et al. [61]	0.299	2.049	0.530	7.927
Meta-analysis (fixed effect)	0.025	1.687	1.067	2.668
